# Overexpression of ZNF488 supports pancreatic cancer cell proliferation and tumorigenesis through inhibition of ferroptosis via regulating SCD1-mediated unsaturated fatty acid metabolism

**DOI:** 10.1186/s13062-023-00421-6

**Published:** 2023-11-20

**Authors:** Qifeng Xiao, Zhongmin Lan, Shuisheng Zhang, Hu Ren, Shunda Wang, Peng Wang, Lin Feng, Dan Li, Chengfeng Wang, Xiaofeng Bai, Jianwei Zhang

**Affiliations:** 1 Pancreatic and gastric surgery department, National Cancer Center/National clinical research center for cancer/Cancer Hospital, Chinese Academy of Medical Sciences and Peking Union Medical College, Beijing, 100021 China; 2 State Key Laboratory of Molecular Oncology, Cancer Hospital, National Cancer Center, Chinese Academy of Medical Sciences and Peking Union Medical College, Beijing, 100021 China

**Keywords:** Pancreatic cancer, ZNF488, Ferroptosis, SCD1, Gemcitabine

## Abstract

**Background:**

Pancreatic cancer is a malignancy with high mortality. Once diagnosed, effective treatment strategies are limited and the five-year survival is extremely poor. Recent studies have shown that zinc finger proteins play important roles in tumorigenesis, including pancreatic cancer. However, it remains unknown on the clinical significance, function and underlying mechanisms of zinc finger protein 488 (ZNF488) during the development of pancreatic cancer.

**Methods:**

The clinical relevance of ZNF488 and stearoyl-CoA desaturase 1 (SCD1) was examined by analyzing the data from The Cancer Genome Atlas (TCGA) and immunohistochemical staining of the tissue microarray. Gain-of-function and loss-of-function experiments were performed by transfecting the cells with overexpressing lentivirus and siRNAs or shRNA lentivirus, respectively. The function of ZNF488 in pancreatic cancer was assessed by CCK8, colony formation, EdU staining, PI/Annexin V staining and xenografted tumorigenesis. Chip-qPCR assay was conducted to examine the interaction between ZNF488 and the promoter sequence of SCD1. Transcription activity was measured by dual luciferase reporter assay. mRNA and protein expression was detected by qRT-PCR and immunoblotting experiment, respectively. Fatty acid was quantified by gas chromatography mass spectrometry.

**Results:**

ZNF488 was overexpressed in pancreatic cancer samples compared with normal tissues. High expression of ZNF488 predicted the poor prognosis of the patients. In vitro, ZNF488 upregulation contributed to the EuU cooperation, proliferation and colony formation of MIAPaCa-2 and PANC-1 cells. Based on PI/Annexin V and trypan blue staining results, we showed that ZNF488 suppressed the ferroptosis and apoptosis of pancreatic cancer cells. Mechanistically, ZNF488 directly interacted with the promoter sequence of SCD1 gene and promoted its transcription activity, which resulted in enhanced palmitoleic and oleic acid production, as well as the peroxidation of fatty acid. In vivo, ZNF488 overexpression promoted the xenograted tumorigenesis of PANC-1, which was reversed by SCD1 knockdown. Importantly, combination of erastin and SCD1 inhibitors A939572 completely blunted the growth of ZNF488 overexpressed MIAPaCa-2 and PANC-1 cells. Usage of A939572 or erastin recovered the sensitivity of pancreatic cancer cells to the treatment of gemcitabine. Lastly, we found a positive correlation between ZNF488 and SCD1 in pancreatic cancer patients based on TCGA and immunohistochemical staining results.

**Conclusion:**

Overexpression of ZNF488 suppresses the ferroptosis and apoptosis to support the growth and tumorigenesis of pancreatic cancer through augmentation of SCD1-mediated unsaturated fatty acid metabolism. Combination of SCD1 inhibitors, ferroptosis inducers or gemcitabine could be applied for the treatment of pancreatic cancer with overexpression of ZNF488.

**Supplementary Information:**

The online version contains supplementary material available at10.1186/s13062-023-00421-6.

## Background

Pancreatic cancer is an uncommon diagnosed cancer but with the worst prognosis because limited therapeutic strategies can be used for the treatment of this deadly malignancy [1]. Development of pancreatic cancer is caused by tobacco smoking, alcohol, type II diabetes and chronic pancreatitis [2]. During the past decades, a wide range of studies based on genomics, transcriptomics, proteomics and metabonomics contribute to the exploration of molecular events responsible for pancreatic cancer progression [3]. Gain-of-function mutations of oncogenes, such as KRAS, GNAS and CTNNB1, and loss-of-function mutations of tumor suppressor genes, including TP53 and CDKN2A, are found as the most important drivers for pancreatic cancers [4]. However, the poor prognosis of pancreatic cancer patients is not improved by the chemotherapy, targeted therapy and immunotherapy even these therapies have gain prominent success on other malignancies, such as melanoma and lung cancer.

Zinc finger protein (ZNF) family comprises of the most profuse proteins in eukaryotes. ZNF was first discovered as a transcription factor in 1985 [5]. As time passed, increased evidences have shown that ZNF not only have the activity as the transcription factors, but also exhibit important roles in post-transcription and other biology processes [6]. Based on the essential functions of ZNF, dysregulation of ZNF participates in the initiation and progression of carcinogenesis. For example, ZNF play either oncogenic or tumor suppressive roles in the development of hepatocellular carcinoma (HCC) [7]. In addition, ZNF have also been reported to have critical roles in pancreatic cancers. ZNF24 can interact with activating transcription factor 3 (ATF3) and acts as a tumor suppressor protein in pancreatic cancer [8]. On the contrary, ZNF91 contributes to the development of pancreatic cancer through activation of β-catenin signaling [9]. As to ZNF488, overexpression of this protein promotes nasopharyngeal carcinoma (NPC) via induction of collagen IV/FAK/AKT signaling pathway [10]. Another study showed that upregulation of ZNF488 potentiated the invasive ability of NPC through activation of Wnt signaling [11]. However, the clinical significance, function and molecular mechanisms of ZNF488 in pancreatic cancer development remain less unclear.

In this study, we aimed to investigate the clinical significance and biology function of ZNF488 in pancreatic cancer based on TCGA analysis, immunohistochemical staining, gain-of-function, loss-of-function, cell proliferation and xenografted tumorigenesis assays. We also dissected the underlying mechanisms of ZNF488 by using qRT-PCR, dual luciferase reporter, ChIP-qPCR, Annexin V/PI staining and metabolites measurement.

## Results

### High expression of ZNF488 in pancreatic cancers confers poor prognosis of the patients

**Fig. 1:**
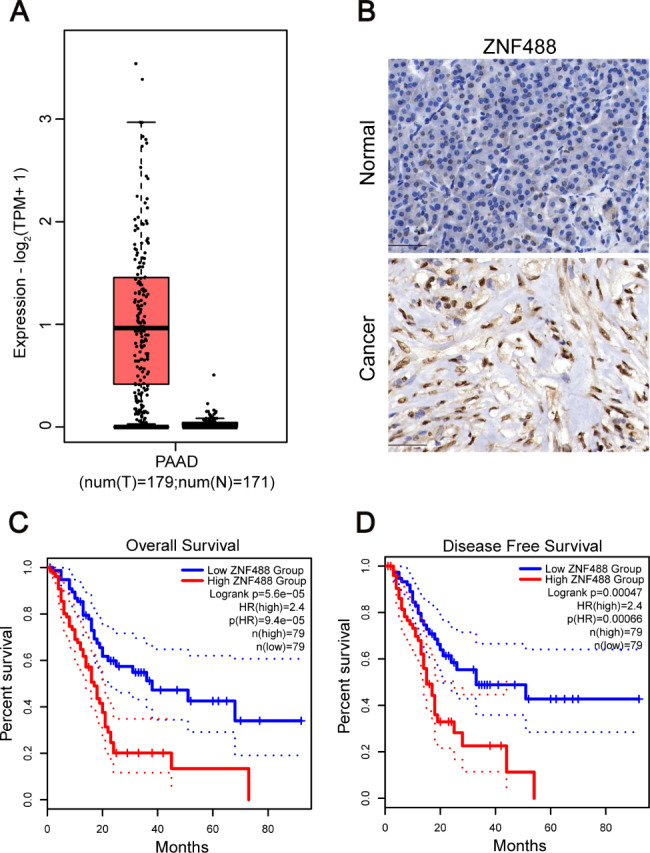
Clinical significance of ZNF488 in pancreatic cancer patients. **A** The mRNA expression of ZNF488 was analyzed in pancreatic adenocarcinoma tissues (n = 179) and normal tissues (n = 171) based on TCGA database. p < 0.01. **B** Immunohistochemical staining of ZNF488 in pancreatic cancer (n = 96) and normal tissues (n = 24). **C, D** The overall and disease free survival was analyzed in the pancreatic adenocarcinoma patients with ZNF488 high expression (n = 79) and low expression (n = 79). p < 0.001

The Cancer Genome Atlas (TCGA) is a public and widely used database for the analysis of gene expression in multiple cancer types. To explore the clinical significance of ZNF488, we compared the expression of ZNF488 in pancreatic cancer and normal tissues. The results showed that ZNF488 was dramatically upregulated in the cancer tissues (Fig. 1A). To further validate the clinical significance in Chinese patients, we performed immunohistochemical staining of ZNF488 and found that ZNF488 was overexpressed in the cancer tissues (Fig. 1B; Table 1). Then, we divided the patients into ZNF488 high expression and low expression group and the patients’ pathologic characteristics were analyzed. We showed that ZNF488 expression was not associated with the age and gender of the patients, while ZNF488 high expression was positively correlated with the stage and T classification of the patients (Table 2). Furthermore, the overall and disease free survival of pancreatic cancer patients with ZNF488 high expression was significantly shorter than those with ZNF488 low expression (Fig. 1C, D). These results indicate that ZNF488 overexpression maybe involved in the progression of pancreatic cancer.

**Table 1:** The expression of ZNF488 in normal tissues and pancreatic cancer tissues by IHC

	Tumor Tissue	Adjacent tissue	χ^2^	p Value
ZNF488 high expression	78	5	32.862	< 0.0001
ZNF488 low expression	18	19		
Total	96	24		

**Table 2:** The relationship between ZNF488 expression and the clinicopathological characteristics of pancreatic cancer patients

	Characteristic	ZNF488 expression		p Value
		High	Low	
Age	< 65	33	8	0.869
	≥ 65	45	10	
Gender	Female	33	10	0.308
	Male	45	8	
Stage I/II/III	I	2	8	< 0.001
	II/III	76	10	
T classification	T0-T1	6	9	< 0.001
	T2-T3	72	9	

### ZNF488 promotes pancreatic cancer cell growth and proliferation

**Fig. 2:**
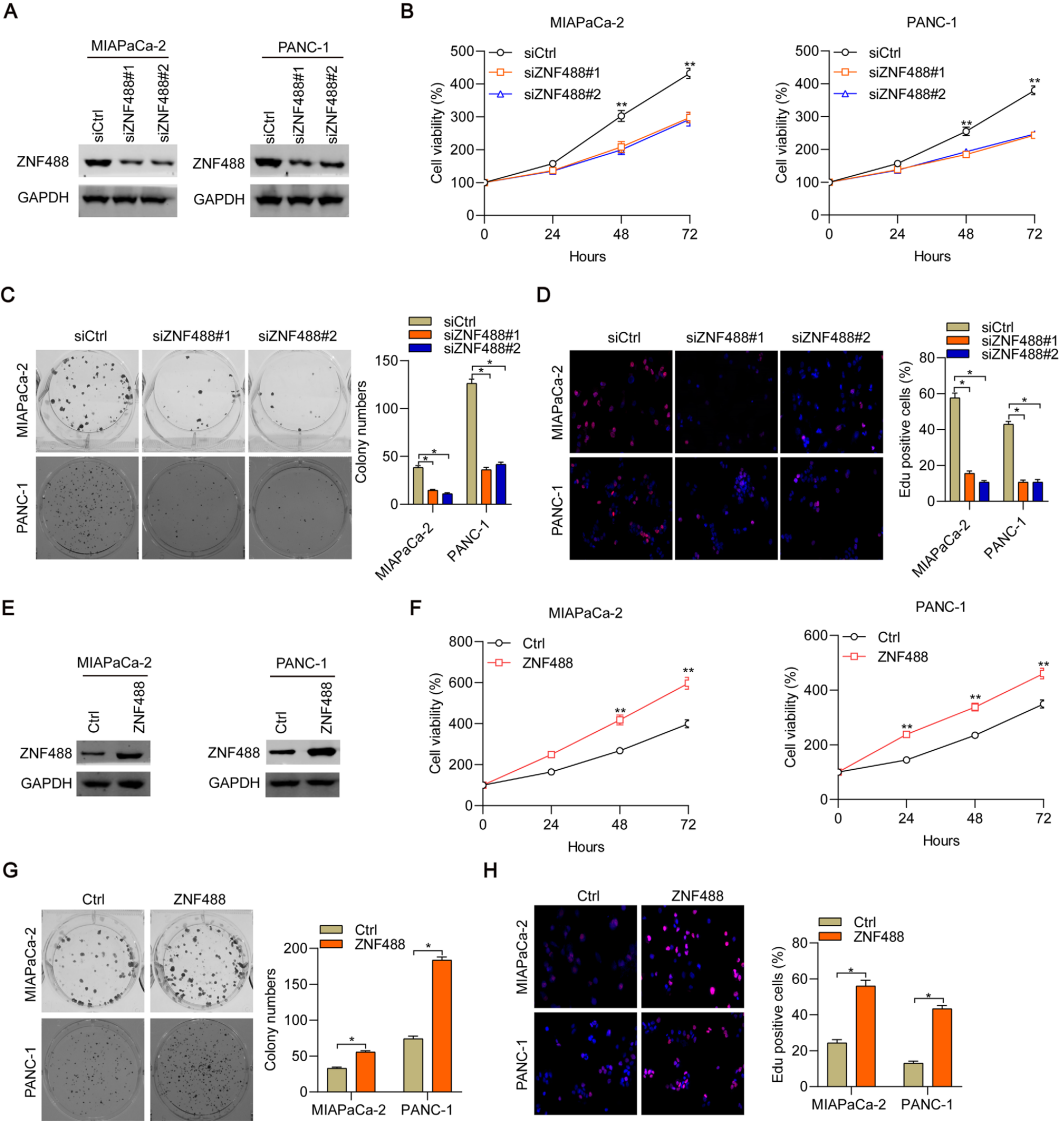
ZNF488 promotes cell proliferation, colony formation and EdU cooperation. **A** siCtrl, siZNF488#1 and siZNF488#2 MIAPaCa-2 and PANC-1 cells were subjected to immunoblotting analysis with indicated antibodies. **B, C** Cell proliferation and growth were detected by CCK-8 and colony formation assay in siCtrl, siZNF488#1 and siZNF488#2 MIAPaCa-2 and PANC-1 cells. **D** EdU staining was analyzed in siCtrl, siZNF488#1 and siZNF488#2 MIAPaCa-2 and PANC-1 cells. **E** Ctrl and ZNF488 overexpressed MIAPaCa-2 and PANC-1 cells were subjected to immunoblotting analysis with indicated antibodies. **F**, **G** Cell proliferation and growth were detected by CCK-8 and colony formation assay in Ctrl and ZNF488 overexpressed MIAPaCa-2 and PANC-1 cells. **H** EdU staining was analyzed in Ctrl and ZNF488 overexpressed MIAPaCa-2 and PANC-1 cells. *p < 0.05. **p < 0.01

To explore the function of ZNF488 in pancreatic cancer, we constructed overexpressing and knockdown cells by using lentivirus and siRNAs. Firstly, we transfected MIAPaCa-2 and PANC-1 cells with siRNAs against negative control and ZNF488. The knockdown efficacy was demonstrated by immunoblotting results (Fig. 2A). Then we subjected the cells to CCK-8 analysis of cell proliferation. We found that ZNF488 downregulation blunted the proliferation ability of MIAPaCa-2 and PANC-1 cells (Fig. 2B). Colony formation assay confirmed that ZNF488 knockdown suppressed the growth of cancer cells (Fig. 2C). To confirm the effect of ZNF488 on pancreatic cancer cell proliferation, we stained the cells with DAPI and EdU, a predictor for DNA replication and cell proliferation. We showed that ZNF488 silencing diminished the percentage of EdU positive cells (Fig. 2D). Next, we overexpressed ZNF488 in the cells and the ectopic expression of ZNF488 was demonstrated by immunoblotting results (Fig. 2E). On the contrary to loss-of-function of ZNF488, its overexpression led to accelerated proliferation and colony formation of MIAPaCa-2 and PANC-1 cells (Fig. 2F, G). When staining the cells with less EdU, ZNF488 significantly strengthened the percentage of EdU positive cells (Fig. 2H). Collectively, ZNF488 functions as an oncogene in pancreatic cancer.

### ZNF488 supports pancreatic cancer cell viability through inhibition of ferroptosis and apoptosis

**Fig. 3:**
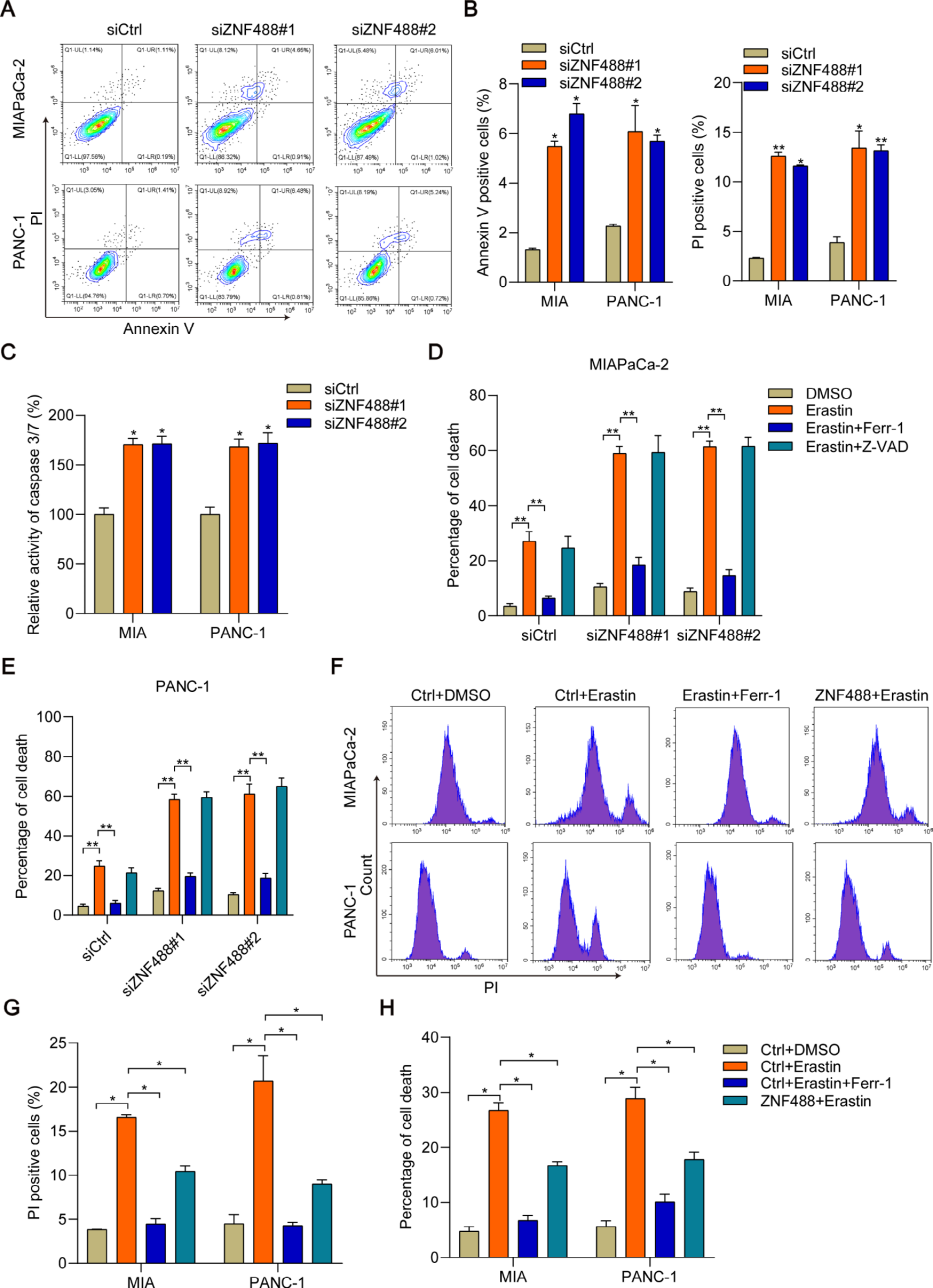
ZNF488 regulates apoptosis and ferroptosis in pancreatic cancer cells. **A, B** siCtrl, siZNF488#1 and siZNF488#2 MIAPaCa-2 and PANC-1 cells were subjected to PI and Annexin V staining which was analyzed on flow cytometry. **C** Caspase 3/caspase 7 activity was measured. **D, E** siCtrl, siZNF488#1 and siZNF488#2 MIAPaCa-2 and PANC-1 cells were treated with DMSO, erastin (5 µM), combining with or without ferrostatin-1 (2 µM) or Z-VAD-FMK (8 µg/ml). Cell viability was analyzed by trypan blue. **F-H** Ctrl and ZNF488 overexpressing MIAPaCa-2 and PANC-1 cells were treated with DMSO or erastin (5 µM), combining with or without ferrostatin-1 (2 µM). Then the cells were subjected to PI staining and trypan blue staining. *p < 0.05. **p < 0.01

To dissect whether ZNF488 regulates cell death of pancreatic cancer cells, we stained the pancreatic cancer transfected with siCtrl or siZNF488 with PI and Annexin V. Flow cytometry analysis showed that ZNF488 knockdown moderately enhanced the percentage of Annexin V positive cells (Fig. 3A, B). In addition, ZNF488 silencing promoted the activity of caspase 3/caspase 7 (Fig. 3C), suggesting that ZNF488 suppresses apoptosis. Obviously, ZNF488 downregulation increased the percentage of PI positive cells in both MIAPaCa-2 and PANC-1 cells (Fig. 3A, B). Recent studies have shown that cell ferroptosis could be marked by PI staining. Thus, we hypothesized that ZNF488 regulated not only apoptosis but also ferroptosis. To address this question, we treated the siCtrl, siZNF488#1 and siZNF488#2 cells with ferroptosis inducer erastin, combining with or without ferroptosis inhibitor ferrostatin-1 and apoptosis inhibitor Z-VAD-FMK. We observed that erastin dramatically induced cell death in siCtrl, siZNF488#1 and siZNF488#2 cells (Fig. 3D, E). Importantly, ZNF488 knockdown promoted the cytotoxicity of erastin in both cells (Fig. 3D, E, siCtrl + erastin vs. siZNF488#1 + erastin and siZNF488#2 + erastin, p < 0.05). Ferrostatin-1, but not Z-VAD-FMK, reversed the cell death caused by erastin in the cells (Fig. 3D, E). To validate the effect of ZNF488 on ferroptosis, we treated Ctrl cells with DMSO, erastin and ferrostatin-1, and treated ZNF488 overexpressing cells with erastin. The cells were subjected to PI staining analysis on flow cytometry. As expected, erastin treatment increased the percentage of PI positive cells, which could be reversed by ferrostatin-1 (Fig. 3F, G). Interestingly, PI positive cells were also reduced by ZNF488 overexpression under the treatment of erastin (Fig. 3F, G). These results suggested that ZNF488 overexpression had similar effect with ferrostatin-1, indicating the suppressive role of ZNF488 on ferroptosis. Likewise, erastin induced cell death could be totally and partly reversed by ferrostatin-1 and ZNF488 overexpression, respectively (Fig. 3H). Taken together, ZNF488 negatively regulates the apoptosis and ferroptosis in pancreatic cancer cells.

### ZNF488 regulates unsaturated fatty acid metabolism through transcriptional activation of SCD1 in pancreatic cancer cells

**Fig. 4:**
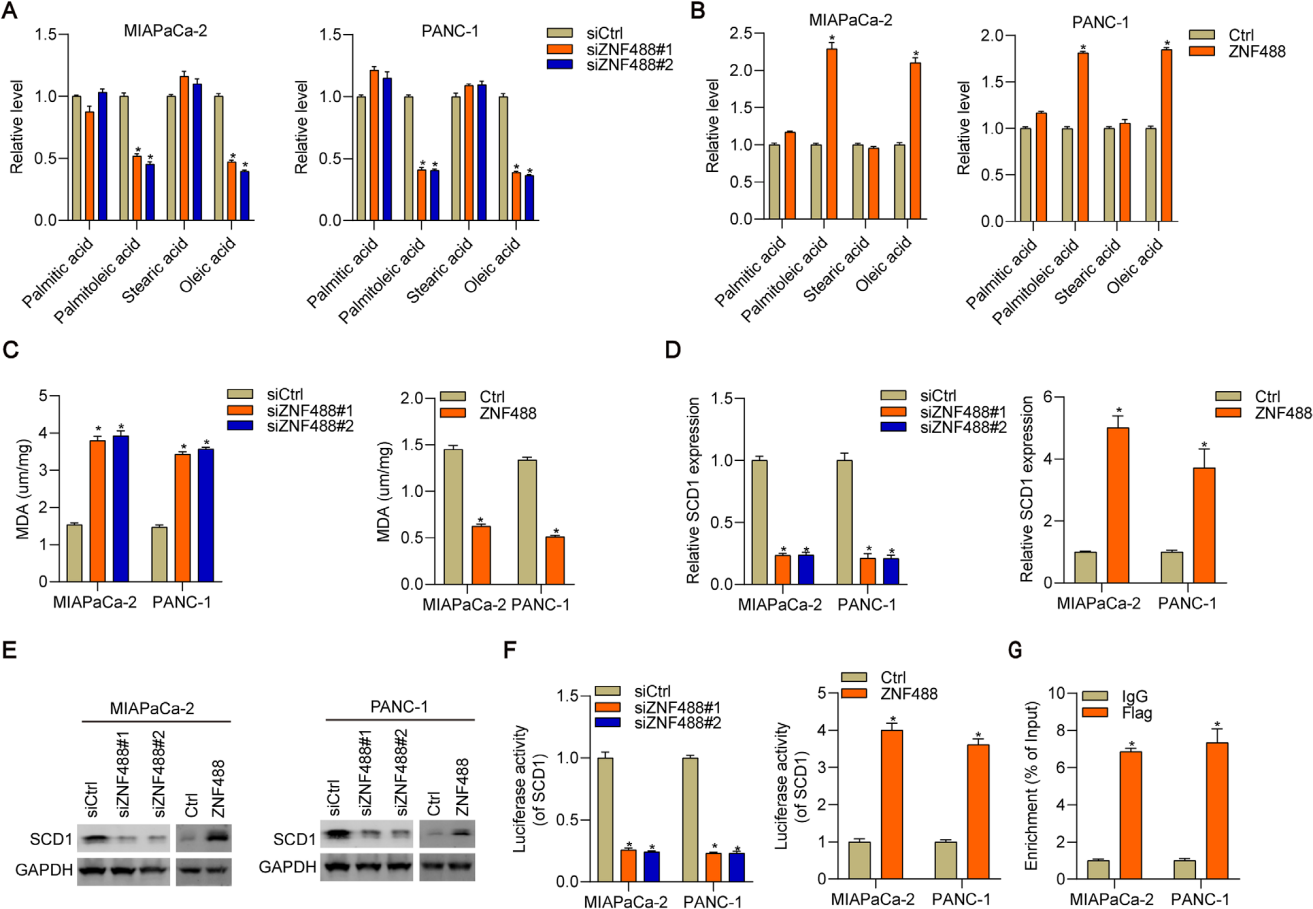
ZNF488 upregulation of SCD1 promotes unsaturated fatty acid metabolism. **A** The abundance of palmitic, palmitoleic, stearic and oleic acid was measured in siCtrl, siZNF488#1 and siZNF488#2 MIAPaCa-2 and PANC-1 cells. **B** The abundance of palmitic, palmitoleic, stearic and oleic acid measured in Ctrl and ZNF488 overexpressing MIAPaCa-2 and PANC-1 cells. **C** MDA levels were checked in ZNF488 knockdown and overexpressing MIAPaCa-2 and PANC-1 cells. **D, E** The mRNA and protein expression of SCD1 was detected in ZNF488 knockdown and overexpressing MIAPaCa-2 and PANC-1 cells. **F** Luciferase reporter assays were performed to check the regulation of ZNF488 on the transcription activity of SCD1 gene. **G** ChIP-qPCR assay was performed to detect the interaction of ZNF488 with the promoter sequence of SCD1. *p < 0.05. **p < 0.01

Ferroptosis is highly correlated with fatty acid metabolism. Thus, we subjected siCtrl, siZNF488#1 and siZNF488#2 MIAPaCa-2 cells to fatty acid profiling via gas chromatography mass spectrometry. We found that ZNF488 downregulation decreased the cellular abundance of palmitoleic and oleic acid, but had no effect on the levels of other fatty acid, such as palmitic acid and stearic acid (Fig. 4A). Consistently, ZNF488 knockdown also inhibited the production of palmitoleic and oleic acid in PANC-1 cells (Fig. 4A). By contrast, ZNF488 ectopic expression enhanced the cellular abundance of palmitoleic and oleic acid in both cells (Fig. 4B). Since ferroptosis is closely associated with the peroxidation of fatty acid, we assessed MDA levels and found that ZNF488 knockdown enhanced fatty acid peroxidation, while ZNF488 overexpression had opposite effects in both cells (Fig. 4C). These results indicate that ZNF488 regulates fatty acid metabolism in pancreatic cancer cells. The synthesis of palmitoleic and oleic acid and fatty acid peroxidation were regulated by SCD1. Then, we tested whether ZNF488 regulated the expression of SCD1. qRT-PCR and immunoblotting results showed that ZNF488 positively regulated the mRNA and protein expression of SCD1 in MIAPaCa-2 and PANC-1 cells (Fig. 4D, E). Dual luciferase reporter assay showed that ZNF488 promoted the transcription activity of SCD1 promoter (Fig. 4F). Furthermore, ChIP-qPCR results showed that ZNF488 directly interacted with the promoter sequence of SCD1 (Fig. 4G). Our results demonstrate that ZNF488 transcriptional upregulation of SCD1 enhances unsaturated fatty acid production and suppresses fatty acid peroxidation in pancreatic cancer cells.

### ZNF488 promotes xenografted tumorigenesis via upregulation of SCD1

**Fig. 5:**
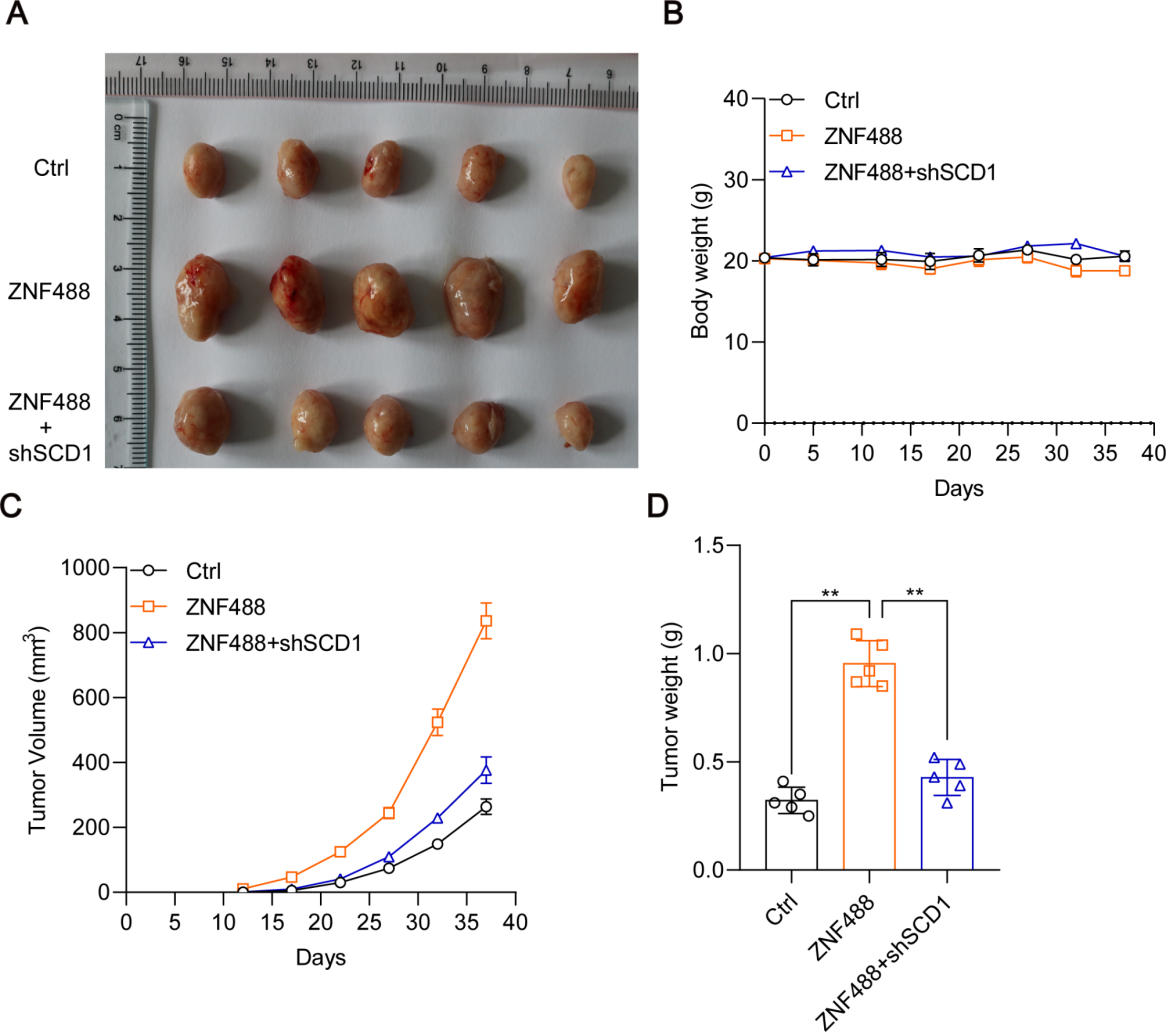
ZNF488 upregulation of SCD1 promotes xenografted tumorigenesis. Ctrl, ZNF488 overexpressing and ZNF488 + shSCD1 PANC-1 stable cells lines were subcutaneously implanted into the nude mice for tumorigenesis. **A** The macroscopical images of tumors collected from the nude mice 37 days after cells implantation. **B, C** Tumor volume and body weight of the mice were monitored during tumorigenesis. **D** Tumors were weighted after sacrificing the mice. *p < 0.05. **p < 0.01

To explore the in vivo role of ZNF488 and SCD1 in pancreatic cancer, we infected the PANC-1 cells with empty Ctrl lentivirus, ZNF488 overexpressing lentivirus and ZNF488 overexpressing with SCD1 knockdown lentivirus. Stable cell lines were selected by puromycin incubation and subcutaneously implanted into the right arm of 6-week-old female nude mice. We found that the body weight of the mice kept similar among the three groups (Fig. 5B). ZNF488 significantly accelerated the tumor initiation and progression of PANC-1 cells, which were alleviated by SCD1 knockdown (Fig. 5A, C, D). Therefore, ZNF488 contributes to pancreatic cancer development through upregulating SCD1.

### SCD1 inhibitor promotes the sensitivity of erastin and gemcitabine in pancreatic cancer cells with ZNF488 overexpression

**Fig. 6:**
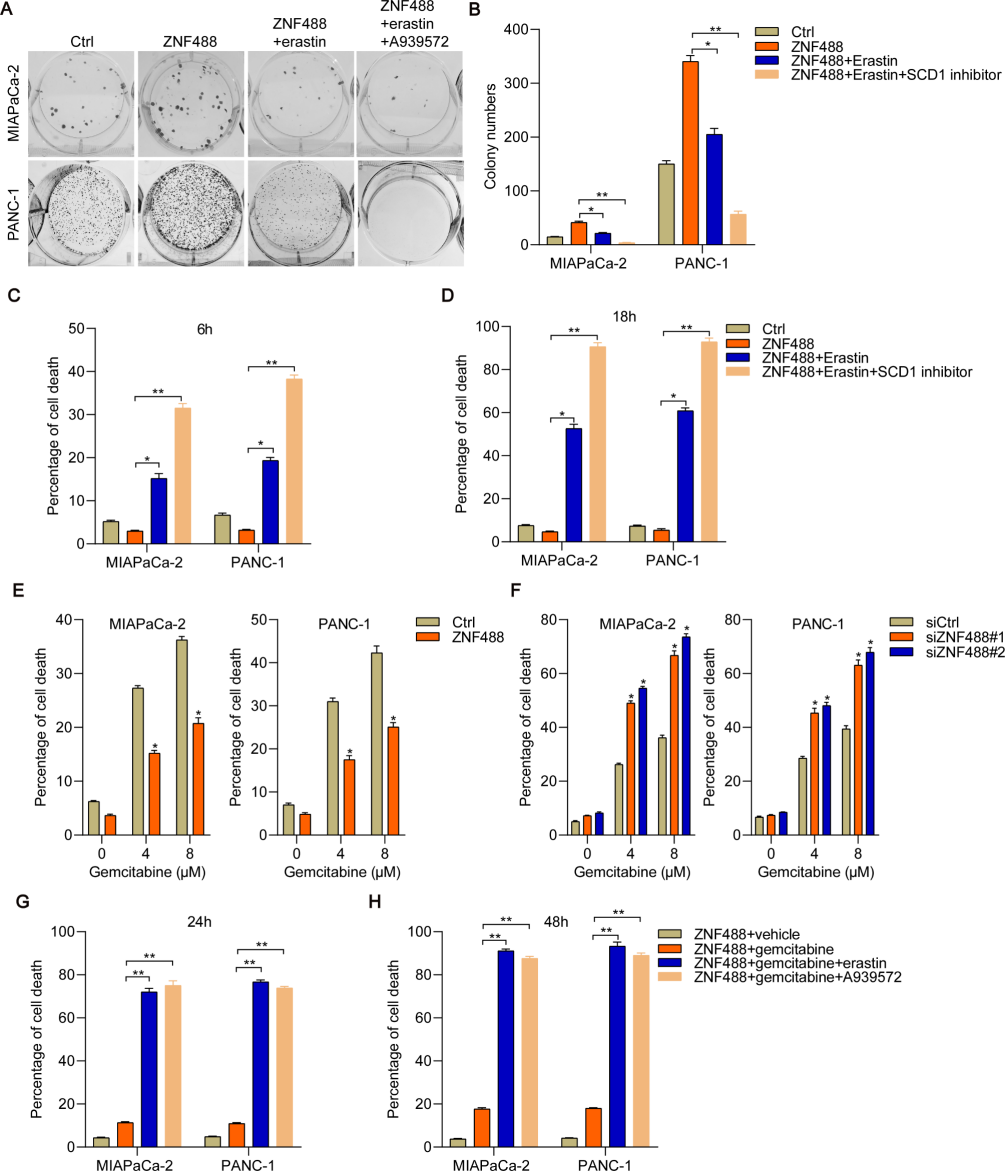
Inhibition of SCD1 promotes the sensitivity of pancreatic cancer cells to the treatment of erastin and gemcitabine. **A-D** Ctrl and ZNF488 cells were seeded in 6-well plates. Then ZNF488 overexpressing cells were treated with erastin alone or combined with SCD1 inhibitors. **A, B** Colony formation was checked. **C, D** After treatment for 6 and 18 h, cell viability was measured by trypan blue staining. **E, F** ZNF488 overexpressing or knockdown pancreatic cancer cells were treated with different concentrations (0, 4 and 8µM) of gemcitabine for 48 h. Cell viability was assessed by trypan blue staining. **G, H** ZNF488 overexpressing pancreatic cancer cells were treated with gemcitabine alone (4µM) or combining with SCD1 inhibitors A939572 (50 nM) and erastin (3µM) for 24 and 48 h. Cell viability was detected by trypan blue staining. *p < 0.05. **p < 0.01

To explore the treatment potential of SCD1 axis and ferroptosis for pancreatic cancers with highly expressed ZNF488, we applied SCD1 inhibitors A939572 and erastin to treat pancreatic cancer cells with overexpression of ZNF488. The results showed that combination of erastin and SCD1 inhibitors synergistically induced the death of pancreatic cancer cells with highly expressed ZNF488 (Fig. 6A-D), suggesting that SCD1 inhibitors eliminate the resistance of ZNF488 overexpressed cells to ferroptosis inducers. Gemcitabine is a widely used chemotherapeutic drug for the treatment of pancreatic cancer patients. Since ferroptosis and apoptosis play essential role in the cell death induced by gemcitabine, we predicted that ZNF488 expression could regulate the sensitivity of pancreatic cancer cells to the treatment of gemcitabine. We found that ZNF488 overexpression promoted, while its knockdown reduced the resistance of pancreatic cancer cells to different concentrations of gemcitabine (Fig. 6E, F). Furthermore, SCD1 inhibitors or erastin could promote the sensitivity of gemcitabine treatment in pancreatic cancer cells with overexpression of ZNF488 (Fig. 6G, H), indicating that SCD1 inhibitors and erastin are potential drugs when combining with gemcitabine for the treatment of pancreatic cancer patients with highly expressed ZNF488. Taken together, SCD1 activation by ZNF488 confers the resistance of pancreatic cancer cells to ferroptosis inducer and ferroptosis-associated chemotherapy.

### ZNF488 is positively correlated with SCD1 in pancreatic cancer patients

**Fig. 7:**
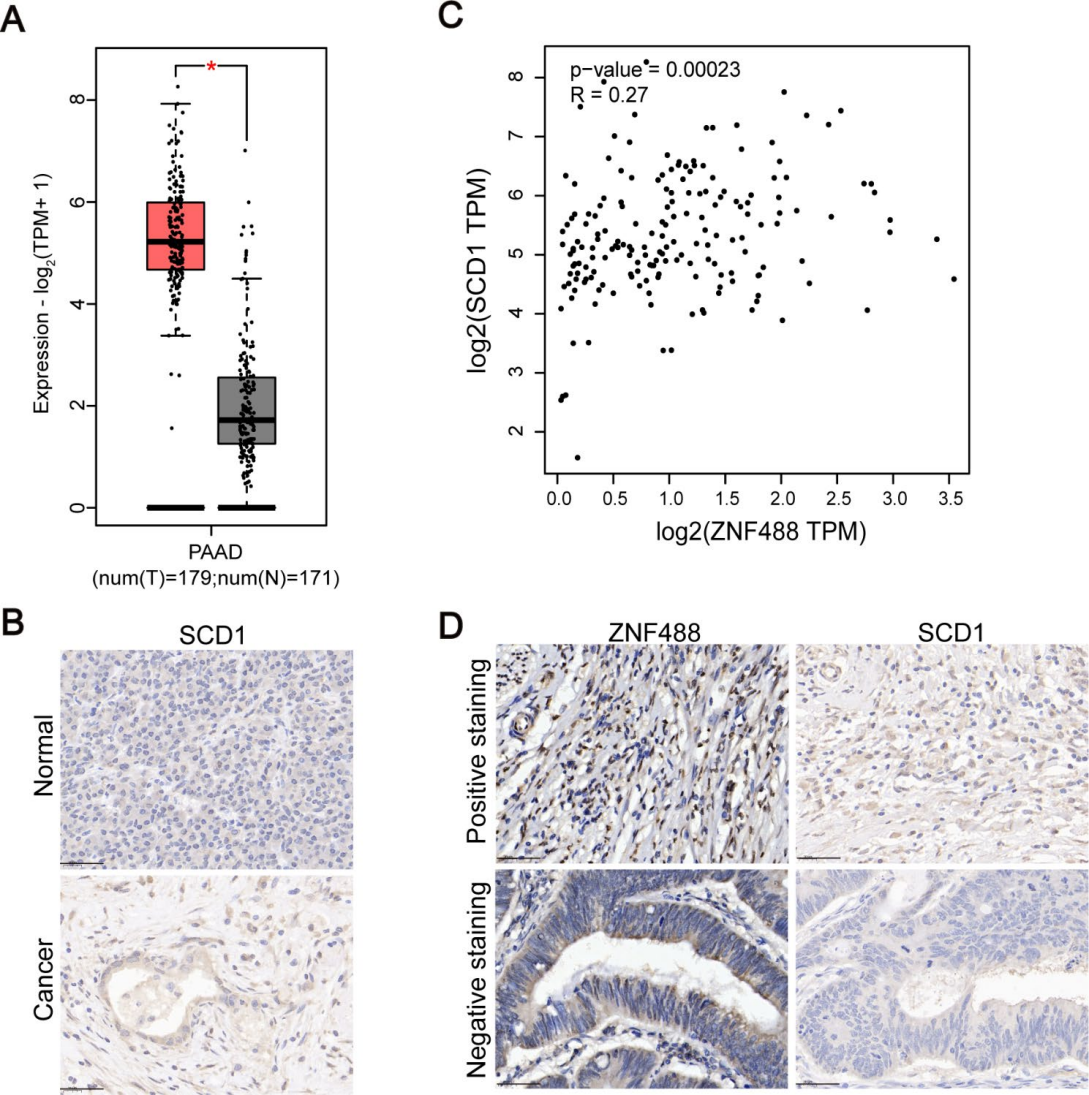
Correlation between ZFN488 and SCD1 in pancreatic cancer patients. **A** Transcript abundance of SCD1 was analyzed in pancreatic cancer and normal tissues based on TCGA data. *p < 0.05. **B** Spearman correlation between ZNF488 and SCD1 was assessed in pancreatic cancer patients based on TCGA data. R = 0.27. p = 0.00023. **C** Immunohistochemical staining of SCD1 in pancreatic cancer (n = 96) and normal tissues (n = 24). **D** Immunohistochemical staining of ZNF488 and SCD1 in pancreatic cancer tissues (n = 52)

Lastly, we investigated the clinical relationship between ZNF488 and SCD1 in pancreatic cancer patients. Based on TCGA data, we showed that SCD1 was dramatically upregulated in pancreatic cancer samples (Fig. 7A). IHC staining confirmed that SCD1 was highly expressed in the cancer samples as compared with normal samples (Fig. 7B). TCGA database showed that there was a positive correlation between ZNF488 and SCD1 in the cancer tissues (Fig. 7C). Furthermore, we checked the protein expression of SCD1 and ZFN488 in the same pancreatic cancer tissues. We found that ZNF488 was positively correlated with SCD1 in the cancer samples (Fig. 7D; Table 3). These results suggest that ZNF488 may positively regulate the expression of SCD1 in pancreatic cancer patients.

**Table 3:** Spearman correlation between ZNF488 and SCD1 in 52 pancreatic cancer tissues by IHC.

	ZNF488	
	*r*_*s*_	*P* value
SCD1	0.632	< 0.0001

## Discussion

In this study, we revealed that ZNF488 was a worse prognostic factor in pancreatic cancer. Overexpression of ZNF488 contributed to the proliferation and tumorigenesis of pancreatic cancer cells, while opposite effect was observed after ZNF488 knockdown. Mechanistically, ZNF488 transcriptionally activated SCD1, leading to enhanced production of monounsaturated fatty acid and suppressed peroxidation of fatty acid. This phenomenon could explain why ferroptosis was inhibited by ZNF488. Furthermore, ZNF488 overexpression conferred the resistance of pancreatic cancer cells to treatment of gemcitabine, which induced cell death partly through ferroptosis.

ZNF488 gene locates in 10q11.22 and the encoded protein belongs to zinc finger protein (ZNF) family, which consists of the largest transcription factors in eukaryotes. A previous study showed that ZNF488 participated in regulating the dorsoventral patterning of spinal tube in chicken [12]. In addition, ZNF488 had important function in the differentiation of oligodendrocyte [13]. Since ZNF can regulate the expression of various genes, abnormal expression of ZNF plays an essential role in regulating cellular metabolism, proliferation, apoptosis, and tumorigenesis [7, 14, 15]. Recently, ZNF488 was reported as an oncogenic protein in nasopharyngeal carcinoma growth and invasion by activation of collagen IV/FAK/AKT/Cyclin D1 pathway and epithelial mesenchymal transition [10, 11]. In cervical cancers, ZNF488 was overexpressed in the patients and it contributed to the malignant growth of the cancer cells via regulating MEK/ERK signaling pathway [16]. Despite that ZNF488 was also found to be involved in pancreatic cancer cell invasion [17, 18], the diagnostic potential, biology function and underlying mechanisms of ZNF488 needed further studies to dissect. In this study, we showed that ZNF488 overexpression conferred poor prognosis of pancreatic cancer patients. ZNF488 upregulation suppressed the apoptosis and ferroptosis of pancreatic cancer cells, thus leading to potentiated cancer cell proliferation and growth in vitro and in vivo. Furthermore, ZNF488 overexpression also promoted the resistance of pancreatic cancer cells to gemcitabine treatment. Thus, ZNF488 overexpression had remarkable significance for the development and clinical treatment of pancreatic cancer patients.

Unlike apoptosis, ferroptosis is a cell death type closely associated with metabolic dysregulation, such as lipid peroxidation [19]. Suppressed ferroptosis contributes to the growth, migration, invasion and development of various cancers [20, 21]. Mechanistically, ferroptosis is regulated by kinds of oncogenes or tumor suppressor genes, such as Kras, PTEN, AKT, and TP53 [22–26]. Invasive explorations have been conducted to evaluate the potential of ferroptosis inducers as the therapeutic strategies for cancer patients [27, 28]. These findings suggest that exploring the controller of ferroptosis may help the pharmacies and oncologists with the investigation and application of ferroptosis inducers. In this study, we observed that ZNF488 overexpression not only suppressed the apoptosis, but also had higher inhibitory effect on the ferroptosis of pancreatic cancer cells. ZNF488 overexpression suppressed, while its knockdown promoted the cell death induced by ferroptosis inducer erastin, indicating that ZNF488 exhibits a ferroptosis inhibitor function similar with ferrostatin-1. Because ferroptosis is closely related to lipid metabolism, we also checked lipid peroxidation and found that the production of MDA was suppressed by ZNF488. Furthermore, ZNF488 promoted the synthesis of palmitoleic and oleic acid. These results indicate that ZNF488 inhibits ferroptosis at least partly through regulating lipid metabolism.

Stearoyl-CoA desaturase 1 (SCD1) gene encodes an enzyme that catalyzes the synthesis of palmitoleic and oleic acid [29]. Overexpression of SCD1 contributes to the progression of malignant tumors by regulating ferroptosis, apoptosis and autophagy [30–32]. The expression of SCD1 is regulated by various upstream proteins during carcinogenesis. For example, SCD1 is one of the most important downstream targets for the transcription factor sterol regulatory element-binding protein 1 (SREBP1). The SREBP1/SCD1 axis promotes the development of hepatocellular carcinoma [33], prostate cancer [26], colorectal cancer [34], gastric cancer [35], and pancreatic cancer [36]. Tumor suppressor gene FBW7 negatively regulated the expression of SCD1 through downregulating nuclear receptor subfamily 4 group A member 1 (NR4A1). The FBW7-NRA41-SCD1 axis plays an important role in regulating the apoptosis and ferroptosis of pancreatic cancer cells [32]. Here, we demonstrated that ZNF488 directly interacted with the promoter sequence of SCD1 and enhanced the transcription activity of SCD1. The mRNA and protein levels of SCD1 were potentiated by ZNF488. Rescue experiments demonstrated that ZNF488 overexpression promoted the xenografted tumor development of pancreatic cancer cells, while knockdown of SCD1 largely reversed the phenotype. Using SCD1 inhibitors could reverse the resistance of ZNF488 overexpressing cells to the treatment of erastin. These results suggest that ZNF488 contributes to pancreatic carcinogenesis and erastin resistance through transcriptional activation of SCD1. However, downregulation of SCD1 could only partly reduce ZNF488-triggered tumor growth, indicating that there might be other molecular mechanisms. Whether SREBP1 participates in ZNF488/SCD1 axis and other downstream targets of ZNF488 should be explored in the future study. A previous study showed that SCD1-mediated ferroptosis and apoptosis not only contributed to the progression of pancreatic cancer, but also played a pivotal role in the sensitivity of gemcitabine in pancreatic cancer [32]. We here found that when ZNF488 overexpression conferred the cells resistant to gemcitabine, this effect could be reduced by SCD1 inhibitors and ferroptosis inducers. Therefore, ZNF488 upregulation of SCD1 and suppression of ferroptosis could be targeted to enhance the chemotherapy effectiveness of pancreatic cancer patients.

## Conclusion

In summary, we provided the first evidence that ZNF488 contributed to pancreatic cancer tumorigenesis through suppression of ferroptosis which was depending on SCD1-mediated fatty acid metabolism. Targeting ZNF488/SCD1 axis, in combination with ferroptosis inducer or gemcitabine could benefit for the patients with highly expressed ZNF488.

## Methods

### Cell culture

Human pancreatic cancer cells PANC-1 and MIAPaCa-2 were purchased from ATCC (Manassas, VA, USA). PANC-1 was a hypertriploid human cell line derived from the pancreatic duct of a 56-year-old, white, male patient with epithelioid carcinoma. MIAPaCa-2 was a hypotriploid human cell line isolated from the pancreas of a 65-year-old, white male patient with carcinoma. The modal chromosome number of PANC-1 and MIAPaCa-2 is 63 and 61, respectively. The cells were maintained in Dulbecco modified Eagle’s medium (DMEM, Hyclone, cat. SH30249.02, Logan, UT, USA), supplied with 10% fetal bovine serum (FBS, Gibco, cat. 10,099,141 C, Grand Island, NY, USA) and 1% penicillin and streptomycin (Corning, cat. 30-002-CI, Corning, NY, USA). The cell culture was kept in a 37^o^C incubator with 5% CO_2_.

## Immunohistochemical staining of ZNF488 and SCD1 in pancreatic cancer patients

Immunohistochemical experiments were conducted in pancreatic cancer tissue microarray, following the protocols as described previously [37]. ZNF488 (21014-1-AP, 1:200) and SCD1 (2794, 1:200) primary antibody was purchased from Proteintech (Chicago, IL, USA) and Cell Signaling Technology (Danvers, MA, USA).

### ZNF488 and SCD1 knockdown

ZNF488 was knocked down by transfecting PANC-1 and MIAPaCa-2 cells with siRNAs using RNAiMAX (Invitrogen, cat. 13778500, Carlsbad, CA, USA). The sequences of siRNAs were as following: siCtrl, 5’-UUCUCCGAACGUGUCACGU-3’; siZNF488#1, 5’-GCACGAAGUGAGCAAAGAA-3’; siZNF488#2, 5’-CCUGAUUGCUGGAACCUAA-3’. 48 h later, knockdown efficacy was checked by immunoblotting and the cells were subjected to other experiments. SCD1 was knocked down by transfecting the cells with lentivirus (Beijing Syngenbio Co., LTD., Beijing, China). 48 h later, stable cell lines were selected by incubating the cells with puromycin.

### ZNF488 overexpression

ZNF488 overexpression was conducted by transfecting the cells with lentivirus. 48 h later, stable cell lines were selected by puromycin. Lentivirus were packaged in 293FT cells after transfecting the cells with pLV-CMV-MCS-EGFP-3Xflag-IRES-Puro, pCMV-dR8.2 and VSV-G by Lipofectamine® 2000 (Invitrogen, cat. 11,668,019). 48 h later, culture supernatant was harvested and subjected to centrifuging at 8,0000×g for 2 h. Overexpression efficacy was examined by immunoblotting assay.

### RNA extraction and quantitative real-time polymerase chain reaction (qRT-PCR)

otal RNA was extracted from PANC-1 and MIAPaCa-2 cells with Trizol (Invitrogen, cat. 15596026CN). The quantity and quality of the RNA were measured by Nanodrop. Reverse transcription of the RNA was performed by using M-MLV reverse transcriptase (Promega, cat. M1705, Madison, WI, USA), according to the manufacturers’ instructions. Subsequently, detection of the cDNA of indicated genes was conducted by qPCR using SYBP Green master mixture on the Bio-rad system. The qPCR primer sequences were as follows: SCD1 forward, 5’-TTCCTACCTGCAAGTTCTACACC-3’, and reverse, 5’-CCGAGCTTTGTAAGAGCGGT-3’; and GAPDH forward, 5’-TGACTTCAACAGCGACACCCA-3’, and reverse, 5’-CACCCTGTTGCTGTAGCCAAA-3. The expression of SCD1 was adjusted to GAPDH.

### Immunoblotting assay

Total proteins were extracted from PANC-1 and MIAPaCa-2 cells using RIPA lysis buffer (Beyotime, cat. P0013B, Shanghai, China), supplied with protease and phosphatase inhibitors cocktail. After checking the protein concentrations by BCA assay kit (Beyotime, cat. P0009), loading buffer was used to adjust the concentration. Then, 30–60 ug of the proteins were separated on 10–12% SDS-PAGE gels, followed by transferring the proteins onto PVDF membranes. The membranes were incubated with 5% skim milk for 1 h at room temperature. After incubating the membranes with indicated primary antibodies at 4℃ overnight, they were washed by PBST for three times and were incubated with indicated secondary antibodies. Lastly, protein signal was examined by SuperSignal West Pico PLUS (Thermo Fisher Scientific, cat. 34,577, Waltham, MA, USA).

### Measurement of cell viability

Cell viability was measured by cell counting kit-8 (CCK-8, Beyotime, cat. C0039) assay and trypan blue staining. For CCK-8 experiment, a total of 3000 cancer cells were seeded into 96-well plates for indicated time. 20 µl CCK-8 reagents were added into each well containing 200 µl culture medium and the plates were maintained at 37^o^C. 3 h later, the OD value at 450 nm was measured on the micro-plate machine. For trypan blue staining, cancer cells were seeded into 6-well plates and were incubated with indicated drugs. Few hours later, the cells were trypsinized and stained with trypan blue, followed by the haemocytometer counting.

### Colony formation assay

A total of 500 PANC1 and MIAPaCa-2 cells were seeded into the 6-well plates. 10 days later, colonies were formed and the cell culture was removed. After washed by PBS for three times, the colonies were fixed by methanol and stained by crystal violet. Subsequently, the plates were washed by clean water and dried at room temperature. Then the colonies were photographed by the camera.

### PI and annexin V staining

PANC1 and MIAPaCa-2 cells were seeded in triplicate in 6-well plates. 2 days later, the cells were re-suspended in FBS free culture medium. After stained by PI and annexin V regent (YEASEN, cat. 40302ES50, Shanghai, China), flow cytometer was used to analyze the PANC1 and MIA cells.

### MDA measurement

Lipid Peroxidation MDA Assay Kit (Beyotime, cat. S0131M) was applied to measure the production of MDA in pancreatic cancer cells. In brief, the cells were collected and subjected to protein quantification by BCA assay kit (Beyotime) and MDA detection, according to the manufacturers’ protocols.

### Dual luciferase reporter and ChIP-qPCR assay

Dual luciferase reporter assay was applied to examine the whether ZNF488 regulated the promoter activity of SCD1. The promoter sequence (-2000 bp) was cloned into pGL3.basic plasmids. PANC1 and MIAPaCa-2 cells were co-transfected with siRNAs or overexpressing plasmids (pCDNA3.1), as well as pGL3.basic containing SCD1 promoter and TK plasmids. The luciferase activity was adjusted to TK.

ChIP-qPCR assay was applied to examine whether ZNF488 interacted with the promoter sequence of SCD1 gene. Briefly, pCDNA3.1 plasmids containing the coding sequence of ZNF488 and flag were transfected in PANC1 and MIAPaCa-2 cells. Cells were lysed and subjected to CHIP experiments using SimpleChIP® Plus Enzymatic Chromatin IP Kit (Cell Signaling, cat. 9005) and qPCR assay, according to the manufacturers’ instructions.

### Fatty acid measurement

ZNF488 knockdown and overexpressing PANC1 and MIAPaCa-2 cells were collected and subjected to mass chromatography type gas chromatography mass spectrometry (GCMS, BIOTREE, Shanghai, China) analysis of fatty acid measurement, according to the manufacturers’ protocols.

### Statistical analysis

Graphpad prism software was used to analyze the data as shown in the Figure. Students’t tests were used to determine the difference between two groups. One-way ANOVA was used to determine the difference when more than two groups. Statistical difference was considered significant when p < 0.05.

### Electronic supplementary material

Below is the link to the electronic supplementary material.

Supplementary Material 1

## Data Availability

All data generated or analyzed during this study are included in this published article.

## Abbreviations

ZNF488: Zinc finger protein 488
SCD1: Stearoyl-CoA desaturase 1
TCGA: The Cancer Genome Atlas
HCC: Hepatocellular carcinoma
ATF3: Activating transcription factor 3
NPC: Nasopharyngeal carcinoma
DMEM: Dulbecco modified Eagle’s medium
FBS: Fetal bovine serum
qRT-PCR: Quantitative real-time polymerase chain reaction
CCK-8: Cell counting kit-8
GCMS: Gas chromatography mass spectrometry
