# Supplementary figures and images for: Overexpression of ZNF488 supports pancreatic cancer cell proliferation and tumorigenesis through inhibition of ferroptosis via regulating SCD1-mediated unsaturated fatty acid metabolism
Source: Biol Direct. 2023 Nov 20;18:77. doi: 10.1186/s13062-023-00421-6 (PMC10658979; doi:10.1186/s13062-023-00421-6)

Original WB results


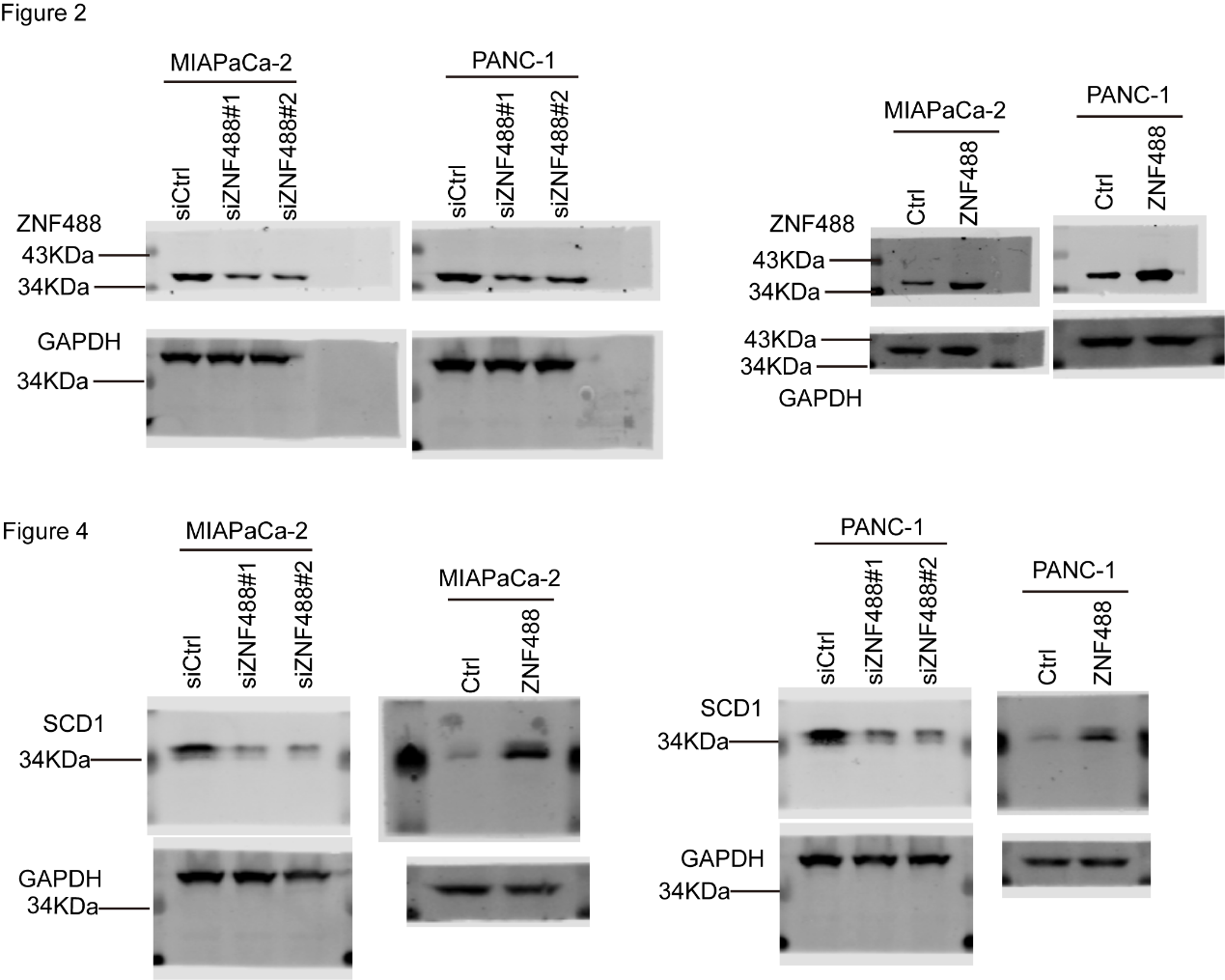

Supplement: Supplementary file 1 — Supplementary Material 1 [file 13062_2023_421_MOESM1_ESM.docx]
